# Possible Role of NRF2 in Cell Response to OZOILE (Stable Ozonides) in Children Affected by Lichen Sclerosus of Foreskin

**DOI:** 10.3390/cimb46090557

**Published:** 2024-08-26

**Authors:** Caterina Saija, Monica Currò, Salvatore Arena, Maria Paola Bertuccio, Fabiola Cassaro, Angela Simona Montalto, Michele Rosario Colonna, Daniela Caccamo, Carmelo Romeo, Pietro Impellizzeri

**Affiliations:** 1 Department of Biomedical and Dental Sciences and Morpho-functional Imaging, University of Messina, 98124 Messina, Italy; caterinasaija93@gmail.com (C.S.); moncurro@unime.it (M.C.); mariapaola.bertuccio@unime.it (M.P.B.); dcaccamo@unime.it (D.C.); 2 Department of Human Pathology of Adult and Childhood “G. Barresi”, University of Messina, 98124 Messina, Italy; arena.salvatore@unime.it (S.A.); fabiola.cassaro@studenti.unime.it (F.C.); angelasimona.montalto@unime.it (A.S.M.); michelerosario.colonna@unime.it (M.R.C.); romeo.carmelo@unime.it (C.R.)

**Keywords:** lichen sclerosus, OZOILE, ozonated oil, nuclear factor erythroid 2–related factor 2 (NRF2), inflammation

## Abstract

Lichen sclerosus (LS) is a chronic inflammatory disease of the skin, and the gold standard for treatment is the use of the very potent topical steroids, but they can have side effects. Previously, we demonstrated that OZOILE (stable ozonides) were effective in children affected by LS, reducing the inflammatory process and stimulating tissue regeneration of the foreskin, showing a similar efficacy to steroid treatment. In this study, the modulation of inflammatory and oxidative stress pathways was evaluated by qRT-PCR and Western blotting in foreskins affected by LS removed from patients untreated or treated with OZOILE or corticosteroid cream formulations for 7 days before circumcision. OZOILE induced a significant increase in NRF2 and SOD2 levels, while it did not produce change in MIF, NF-kB subunits, and MMPs in comparison to untreated foreskins. Conversely, steroid topical treatment produced a significant reduction in the expression of p65, MIF, and MMP9, but it did not cause variation in NRF2 and SOD2 levels. These results demonstrate that the use of OZOILE as cream formulation exhibits effects on NRF2 signaling, and it does not induce NF-κB activation, unlike corticosteroids. On the basis of our biochemical data, further studies evaluating the role of NRF2 signaling cascade are necessary.

## 1. Introduction

Lichen sclerosus (LS) is a chronic skin condition characterized by thinning, inflammation, and scarring of the skin in the genital and anal areas. In children, lichen sclerosus most commonly affects the foreskin of uncircumcised boys. LS was first described by Breisky in 1885 and by Hallopeau in 1887 [1,2]. The disease was referred to with various terms, including lichen sclerosus et atrophicus and kraurosis of the vulva, but in 1976, the International Society for the Study of Vulvovaginal Disease decided to call it lichen sclerosus [3]. LS has been described in adults and children of both sexes and affects women more frequently than men [4,5].

LS in children is relatively uncommon compared to its occurrence in adults, and it is hard to establish its exact incidence due to the rarity of the condition in this age group and potential underdiagnosis because of lack of symptoms. The LS of the foreskin, with an estimated incidence of 35% [6], is a risk factor for acquired phimosis, being associated with 80–90% of cases [7,8]; however, the actual incidence of LS is thought to be clinically underestimated by up to 50% [9].

LS of foreskin, also classified as balanitis xerotica obliterans (BXO) and firstly described by Stuhmer in 1928, is characterized by white atrophic plaques on the foreskin, glans penis, frenulum and meatus, or urethra [5,10].

The etiology of LS is still far from being fully elucidated and likely involves several factors. So far, LS is regarded as an autoimmune disease because of the reported presence of autoantibodies against extracellular matrix protein 1 and BP180 and also because of its reported association with atopy, thyroiditis, alopecia areata, diabetes mellitus type I, vitiligo, pernicious anemia, and celiac and Crohn’s disease. The risk factors highlighted in LS are frequent trauma, the use of certain drugs, and genetic and familial predisposition. The expression of some tissue remodeling-associated genes and an exacerbated oxidative stress have also been described in LS, which may lead to scarring and malignancy [11,12,13].

Our previous study suggested that overexpression of IFN-γ and transglutaminase-2 (TG2) in the foreskin of LS patients can trigger and sustain the inflammatory response to either autoimmune or infective stimuli or chronic irritation. Furthermore, the expression of proteins involved in the maintenance of the structural integrity of the healthy foreskin, such as TG1, TG3, and E-cadherin, were found to be decreased [14].

LS is often misdiagnosed in children, with possible harmful effects and complications such as renal failure and spinocellular carcinoma (SCC) in adult life [15]. Clinical examination alone led to an underestimation of LS in boys [16]. In fact, it is also essential to evaluate the patient’s clinical history, which has a positive predictive value when it includes the presence of autoimmune or allergic disease and having experienced at least one episode of balanitis or a lower urinary tract infection and the lack of improvement after steroids [16].

A recent immunohistological study on the foreskin of boys affected by LS showed an acanthotic epidermis, with infiltration of CD8+ CD57+ lymphocytes, and hyperplasia of keratinocytes overexpressing Ki67 and p53. These findings highlight the risk that pediatric LS can evolve into spinocellular carcinoma in adulthood and suggest the radical circumcision as the best preventive strategy [17].

Thus far, radical circumcision is also considered the definitive treatment of phimosis due to LS [15,18]. Moreover, the use of medications with topical treatments on the foreskin affected by LS, both before and after surgery, has been widely documented [13,18].

The gold standard for the treatment of anogenital LS in adults, girls, and boys, according to updated evidence-based guidelines, is the use of topical steroids such as clobetasol propionate (CP) [13]. However, therapy with corticosteroids may cause side effects such as burning, irritation, hypopigmentation, risk of superinfection, skin atrophy, xerosis, and, rarely, suppression of the hypothalamic–pituitary axis [19].

Both in adults and children, calcineurin inhibitors such as tacrolimus 0.1% ointment or pimecrolimus 1% cream can be considered effective and safe alternatives to CP. However, CP is generally more effective and should continue to be the first-line treatment [20].

In addition, the topical use of vitamin E is regarded as the most effective treatment maintenance after therapy with corticosteroids of vulvar LS [21].

We recently demonstrated that the topical use of ozonated olive oil (OZOILE) in cream formulation before surgical intervention had a favorable effect on patients affected by LS, inducing a significant reduction in the inflammatory status without adverse reactions. Furthermore, OZOILE treatment improved skin structure and integrity, stimulating a faster recovery and promoting the healing process in LS of children or adolescents undergoing circumcision [22,23].

The purpose of this study was to better understand the molecular mechanisms underlying the beneficial effects of topical use of OZOILE. To this aim, we compared its effects versus topical corticosteroid preoperative treatment in children with phimosis and LS who were undergoing circumcision. In particular, we assessed the possible role of the main pathways involved in inflammatory and oxidative stress responses such as nuclear factor κB (NF-κB) and nuclear factor erythroid 2–related factor 2 (NRF2) in response to OZOILE. The first factor is a protein complex that plays a key role in regulating the immune response to infection and inflammation through the expression of several pro-inflammatory cytokines and chemokines and inflammasome activation, and it is also involved in cell proliferation, differentiation, and survival. NRF2 is a transcription factor that plays an important role in cellular defense mechanisms against oxidative stress by regulating the expression of various antioxidant enzymes. It has a general anti-inflammatory effect and also affects the activation of the NF-kB pathway. In addition, we evaluated the expression levels of some targets of these transcription factors, including matrix metalloproteinases (MMPs) and superoxide dismutase 2 (SOD2), which are involved in tissue remodeling and the maintenance of the cellular oxidative–antioxidant balance, respectively.

## 2. Materials and Methods

### 2.1. Materials

OZOILE cream was purchased by Erbagil s.r.l. (Telese Terme, Benevento, Italy). It is a topical formulation containing 23% OZOILE. OZOILE consists of stable ozonides, which are oxygen-rich lipid molecules produced through a patented eco-friendly process. This process involves the reaction of ozone with the olefinic bonds in the fatty acids of +Oil^®^, an organic extra virgin olive oil. The resulting product is a stable form of ozonides. +Oil^®^ is sourced from organic extra virgin olive oil specifically from the Sannio Benevento region in the Campania hinterland, Italy.

### 2.2. Patients Recruitment

For this study, thirty children (9.4 ± 3.6 years; age range 5–15 years) undergoing circumcision because of phimosis and with histological diagnosis of LS were recruited. At the time of surgery, routine biochemical analyses showed normal blood cell counts and C-reactive protein levels.

The patients were divided into three groups, that is, a control group (*n* = 10) including patients untreated before surgery and two groups of patients topically treated once a day for 7 days with creams containing either OZOILE (*n* = 10) or the steroid 0.1% mometasone furoate (*n* = 10).

Foreskin samples obtained during the circumcision procedure were used for biochemical analyses after release of parental informed consent and authorization to use sensitive data of all subjects at the time of admission.

The study protocol was approved by the Ethical Committee of “AOU Policlinico di Messina” (n. 120/2016 of 12 December 2016) and all procedures carried out in accordance with the Helsinki Declaration about ethical standards.

### 2.3. Gene Expression Analysis by Real-Time PCR

RNA isolation and reverse transcription to cDNA as well as quantitative real-time RT-PCR were carried out as previously described [22,23]. The mRNA transcript levels of NRF2, p50, p65, MIF, MMP2, MMP9, and SOD2 were assessed using the primer sequences shown in Table 1.

### 2.4. Western Blotting

Quantification of protein levels of MMP9 and SOD2 as well as NRF2, p65, and laminin B1 in nuclear compartments of foreskin samples was performed by Western blotting and subsequent densitometric analysis of immunoblots, using protocols previously described [22,23].

### 2.5. Power and Sample Size Analysis

The sample size was calculated based on the primary outcome variable, which was the NRF2 mRNA levels. Considering the three groups, a two-sided significance level of 0.05, and a power of 80%, it was determined that a minimum of 27 patients would be required for this study.

### 2.6. Statistical Analysis

Data are expressed as mean ± SD and were analyzed by one-way analysis of variance (ANOVA), followed by the Bonferroni post hoc test using Graph Pad Prism software (version 5.00) (San Diego, CA, USA). *P*-values lower than 0.05 were considered significant.

## 3. Results

The expression levels of NRF2 and NF-κB subunits were examined by real-time PCR and Western blotting in foreskin samples obtained from LS patients untreated or treated with the topical creams, OZOILE, or 0.1% mometasone furoate.

**Figure 1:**
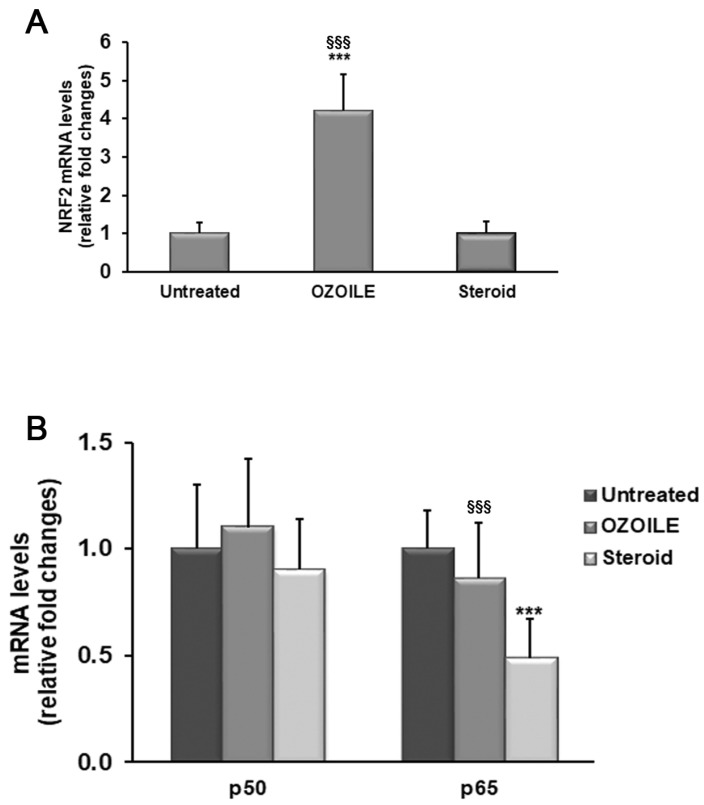
Evaluation of mRNA levels of NRF2 (**A**) and NF-kB subunits (**B**) in foreskin of LS patients untreated (*n* = 10) or topically treated with either OZOILE (*n* = 10) or steroids (0.1% mometasone furoate; *n* = 10). Real-time PCR results (means ± SD) are expressed as relative fold changes in comparison with untreated patients. *** *p* < 0.001 significant differences in comparison with untreated patients; ^§§§^ *p* < 0.001 significant differences in comparison with steroid-treated patients.

**Figure 2:**
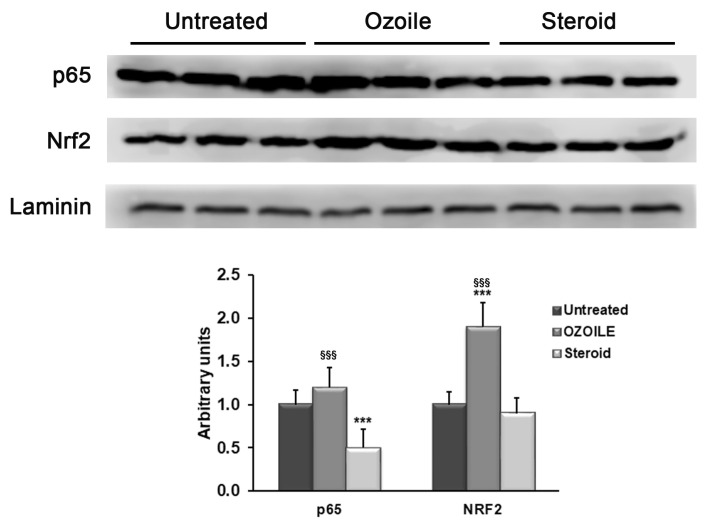
Evaluation of nuclear protein levels of p65 and NRF2 in foreskin of LS patients untreated or topically treated with either OZOILE or steroids (0.1% mometasone furoate). Western blotting results are representative of all tissues analyzed (*n* = 10 per group), and the densitometric analysis data are expressed as the means ± SD of the samples analyzed (*n* = 10 per group). *** *p* < 0.001 significant differences in comparison with untreated patients; ^§§§^ *p* < 0.001 significant differences in comparison with steroid-treated patients.

The treatment with OZOILE induced a significant increase in the NRF2 mRNA levels (4.2 folds) when compared to both untreated or corticosteroid-treated tissues (*p* < 0.001) (Figure 1A), while did not produce changes in the transcript levels of both NF-kB subunits, i.e., p50 and p65, in comparison with foreskin tissues from LS patients without any medications (Figure 1B). Conversely, the corticosteroid cream was effective in reducing the p65 mRNA levels with respect to both untreated and OZOILE-treated tissues (*p* < 0.001), while it was inefficient regarding NRF2 transcription (Figure 1A,B). As shown in Figure 2, these effects were confirmed by the Western blot analysis of protein amounts in nuclear compartment.

**Figure 3:**
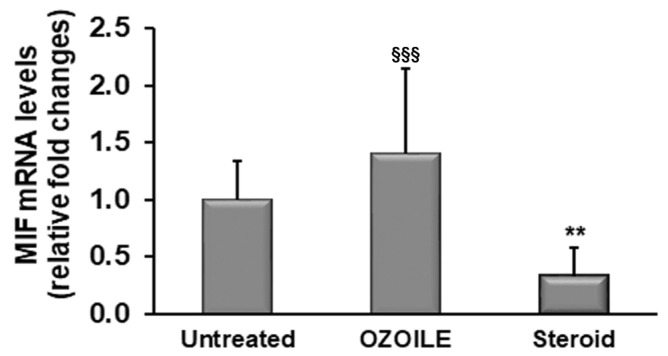
Evaluation of MIF mRNA levels in the foreskin of LS patients untreated or topically treated with either OZOILE or steroids (0.1% mometasone furoate). Real-time PCR results (means ± SD) are expressed as relative fold changes in comparison with untreated patients. ** *p* < 0.01 significant differences in comparison with untreated patients; ^§§§^ *p* < 0.001 significant differences in comparison with steroid-treated patients.

We also evaluated the expression of the macrophage migration inhibitory factor (MIF), which plays a role in inflammation, and we observed a remarkable decrease by 70% in tissues treated with the corticosteroid in comparison to untreated tissues (*p* < 0.01). Also, corticosteroids produced a significant reduction in MIF levels in comparison to foreskins treated with OZOILE (*p* < 0.001). In contrast, OZOILE was not effective in reducing MIF levels (Figure 3).

**Figure 4:**
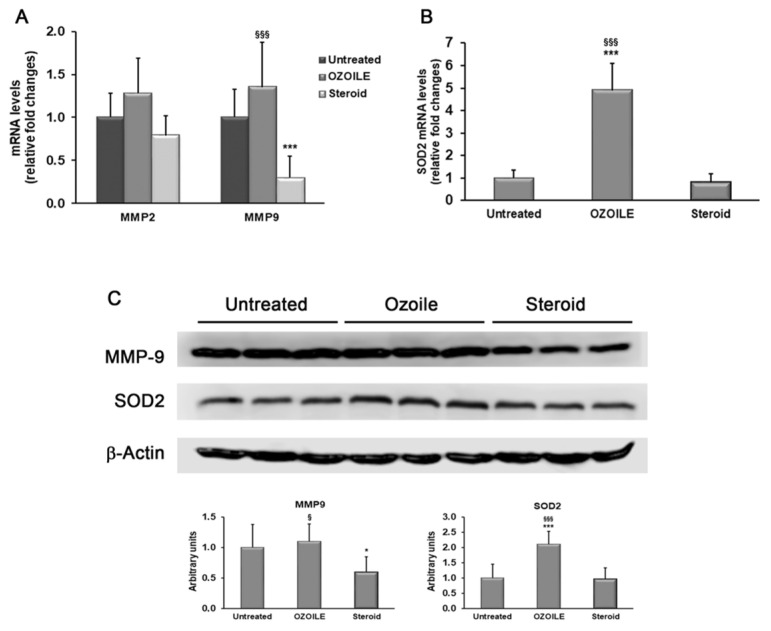
Evaluation of protein levels of MMP9 and SOD2 in the foreskin of LS patients untreated or topically treated with either OZOILE or steroids (0.1% mometasone furoate). Real-time PCR results (means ± SD) are expressed as relative fold changes in comparison with untreated patients (**A**,**B**). The protein amounts were assessed by Western blot analysis (**C**). Western blotting is representative of all tissues analyzed (*n* = 10 for each group), and densitometric analysis data represent the means ± SD of analyzed samples (*n* = 10 for each group). * *p* < 0.05 and *** *p* < 0.001 significant differences in comparison with tissues from untreated patients; ^§^ *p* < 0.05 and ^§§§^ *p* < 0.001 significant differences in comparison with steroid-treated patients.

Finally, we compared the effects of OZOILE and corticosteroid cream formulations on MMP2, MMP9, and SOD2 expression. While OZOILE treatment did not induce variation in the transcript levels of both MMPs, we found a significant decrease by 70% of the MMP9 expression after treatment with 0.1% mometasone furoate compared to untreated tissues (*p* < 0.001) and by about 78% compared to OZOILE treatment (*p* < 0.001) (Figure 4A).

Regarding SOD2 gene, an NRF2 target, its expression was up-regulated in foreskin tissues treated with OZOILE (4.9 folds) (*p* < 0.001) (Figure 4B) when compared with untreated and corticosteroid-treated tissues. Instead, the corticosteroid cream did not produce variations in the transcription levels of SOD2 when compared with foreskin tissues from LS patients without any medications (Figure 4B). As shown in Figure 4C, MMP9 and SOD2 protein amounts changed, according to the real-time PCR results.

## 4. Discussion

LS is a chronic inflammatory dermatosis that may affect the foreskin. In order to obtain a certain diagnosis, several authors believe it is necessary to conduct a histological examination of the foreskin after circumcision in addition to the objective clinical examination [24,25,26]. Possible complications due to a missed diagnosis are meatal stenosis up to renal failure due to urinary obstruction, sexual dysfunction, as well as the potential development of carcinomas in adult life [27,28]. In the literature, it has been reported that meatal stenosis can progress to urethral strictures. If LS progression goes undiagnosed, it can lead to significant morbidity with renal failure due to obstructive uropathy [28,29,30,31,32]. Moreover, it was shown that about 50% of patients suffering from penile SCC also had histological evidence of LS [33].

For this reason, careful and precise follow-up of patients affected by LS of the foreskin must be carried out for a prolonged time [34].

There are various alternative treatments that include formulations for topical use, although surgery with circumcision remains the best choice [6].

Several drugs have been evaluated over the years, but few large-scale studies or RCTs of LS treatment in pediatric patients are available. It was shown that the topical use of corticosteroids before surgery, at the time of surgery, and after surgery [8] can arrest or delay the LS progression [35]. Moreover, Poindexter and coworkers detected a successful evolution of LS in over 90% of cases treated with clobetasol propionate (0.05%) for 2–3 months [36].

A systematic review and meta-analysis using the methodology of the Cochrane Collaboration with the aim to evaluate the effects of various topical interventions for genital LS was published in 2011 [37]. Seven clinical studies met the inclusion criteria defined by the authors and provided little pediatric evidence, with a total of 249 participants who were predominantly adults; the exception was a study recruiting boys [35]. Among the six different treatments reported, an RCT focused on the use of mometasone furoate ointment 0.05% for the LS care of foreskins, and the drug was found to be effective in 40% of the recruited boys. Moreover, the use of topical androgens and progesterone did not seem functional in adults; thus, it was concluded to exclude them from clinical practice in children.

Interesting results were obtained for the tacrolimus 0.1% ointment applied immediately after surgery of fully established LS. Ebert et al. found successful the use of this drug despite the fact that 9% of boys showed a lichenoid inflammatory reaction, which was eradicated by the use of the same drug on the foreskins of boys [38].

Furthermore, the evidence-based guideline proposed by Kirtschig and coworkers reported the use of mometasone and clobetasol for the anogenital treatment in boys only [13].

Despite these results, a moderate and balanced application of corticosteroids according to the damage extension is required because of the unwanted side effects of a long-term treatment, including skin atrophy, xerosis, hypopigmentation, burning, irritation, and, rarely, suppression of the hypothalamic–pituitary axis [19].

Moreover, the treatment of penile LS with clobetasol propionate has been shown to be safe and effective despite potentially triggering latent viral infections, i.e., by human papillomavirus [39]. In addition, the treatment of children with corticosteroids generates anxiety in their parents, a behavior classified as “corticosteroid phobia”, thereby reducing the compliance to the prescriptions of dermatologists and pediatricians [40]. For this reason, other formulations have been considered for LS treatment, either alone or in combination with corticosteroids, to reduce potential undesirable effects. Avocado and soybean extracts have been employed as anti-inflammatory, anti-fibrotic, emollient, and lenitive treatments and are considered effective alternatives in symptoms and signs of mild-to-moderate vulvar LS [41].

Topical formulations based on ozonated olive oil have been shown to be nontoxic and effective on epithelial tissue [42]; thus, OZOILE could represent an alternative to pharmacological therapy.

As previously reported, OZOILE appeared to be effective on inflammation in patients affected by LS, showing beneficial effects similar to corticosteroids [23]. OZOILE is a pool of molecules obtained through a patented process that leads to the formation of stable ozonides. Thus, stable ozonides are a class of organic compounds with an endoperoxide structure produced by the addition of ozone to the olefinic double bonds of the unsaturated fatty acids in extra virgin olive oil. The mechanism of action of these compounds involves the release of molecular oxygen with the formation of oxidative species at low concentrations, which act as regulators of important molecular pathways [43]. Therefore, the moderate oxidative stress generated by stable ozonides, acting on RedOx balance, stimulates the activation of cellular antioxidant defense systems and the production of growth factors [44,45] in order to promote wound healing, tissue repair, and anti-inflammatory responses [42].

In previous studies, we evaluated the action of OZOILE on foreskins removed from LS patients. The results showed a significant reduction in mRNA levels of several markers of inflammation, such as IL-1β, TNF-α, INF–γ, transglutaminase 2, and NOS2, supporting its possible use in LS-affected foreskin as an anti-inflammatory formulation that also exerts beneficial effects against nitrosative stress [22].

Of note, previous results comparing the effects of OZOILE versus 0.1% mometasone furoate demonstrated that the preoperative topical treatment of children affected by LS of the foreskin with OZOILE for 7 days showed similar efficacy to 0.1% mometasone furoate in reducing the inflammatory status, as evidenced by the decreased expression of the pro-inflammatory cytokines TNF-α and IL-1β in treated foreskins [23].

It has been widely demonstrated that corticosteroids have the ability to negatively modulate the expression of several proinflammatory genes by either directly binding to GR elements in gene promoters or indirectly by inhibition of NF-κB, a protein complex that plays a key role in regulating the innate and adaptive immune response to infection and inflammation through the expression of several pro-inflammatory cytokines and chemokines and inflammasome activation. NF-κB is also involved in cell proliferation, differentiation, and survival [46,47,48,49]. Also, the mometasone furoate has been shown to reduce TNFα expression acting on NF-κB [50,51,52].

Regarding the mechanism of action of ozonides, it was also postulated that inducing a moderate oxidative stress can repress inflammatory cascade throughout NF-κB inhibition [53].

**Figure 5:**
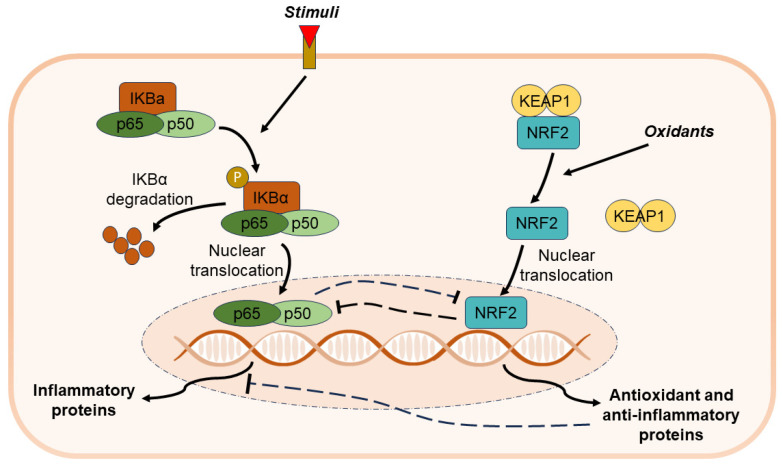
NF-κB and NRF2 pathways activation. The dotted lines highlights the possible link between NF-κB and NRF2 pathways in response to a stimuli.

In addition, several reports hypothesized that the anti-inflammatory effects of therapeutic concentration of ozone involve the activation of NRF2 [53,54,55], the master transcriptional regulator of a multitude of antioxidant-responsive element (ARE) genes [56], which encode for proteins involved in a multitude of vital biological functions, including oxidative stress response and inflammation [57,58]. NRF2 modulates inflammation through multiple mechanisms, such as the regulation of redox homeostasis and the suppression of pro-inflammatory genes, either directly preventing gene transcription of pro-inflammatory cytokines IL-6, IL-1β, and TNF-α [59,60] or through the inhibition of the transcriptional activity of NF-κB. On the other hand, NRF2 activity as well as the expression of its downstream target genes could be interrupted through NF-κB induction (Figure 5) [60,61].

In 2014, the in vivo activation of NRF2 pathway after low dose ozone/oxygen exposure was reported for the first time. In peripheral blood mononuclear cells (PBMC) of healthy volunteers, the in vivo injection of medical-grade ozone gas (major auto-hemotherapy, MAH) determined the induction of NRF2 and the consequent increase in superoxide dismutase and catalase activities, which contributed to favor cell survival [62].

Also, in rats with adenine-induced chronic kidney disease, systemic O3 administration reduced renal injury through the stimulation of NRF2 and the inhibition of NF-κB transcriptional activity, resulting in the up-regulation of antioxidant enzymes and the down-regulation of inflammatory cytokines in the kidney [63]. Moreover, the induction of NRF2 pathways by the application of ozonated saline intervened in wound closure in an in vitro human keratinocyte model of wound healing [64].

In our study, we found that OZOILE and corticosteroid cream formulations act via different molecular pathways in the foreskin of patients affected by LS. According to the literature data, MF induced a significant decrease in the expression of NF-κB subunit p65, while it was not effective on NRF2. In contrast, OZOILE produced a significant increase in NRF2 expression but did not produce changes in the expression levels of NF-κB subunits.

Although NRF2 has been reported to lead to NF-κB inhibition, in the present study, the OZOILE induction of NRF2 was not associated with NF-κB changes, confirming the evidence of our previous study [22]. Our hypothesis is that the short OZOILE treatment in recruited patients undergoing circumcision is not enough to reduce NF-κB activation; maybe a prolonged treatment of foreskins affected by LS could lead to changes in both NRF2 and NF-κB. However, these results indicate that the down-regulation of inflammatory cytokines observed in OZOILE-treated foreskin affected by LS [22,23] could be associated with the activation of the NRF2 pathway.

To further characterize the molecular mechanism underlying OZOILE action, we also explored the role of MIF and MMPs in response to OZOILE and MF treatments.

MIF is a pivotal cytokine involved in inflammation and immune regulation, with potential therapeutic implication. It was reported that NF-κB and MIF collaborate synergically in the modulation of inflammatory processes because the MIF promoter presents two functional binding sites for NF-κB, and on the other hand, MIF itself promotes the activation of NF-kB cascade [65]. MIF is able to promote the activation of macrophages and T cells with the consequent secretion of more proinflammatory cytokines and chemotactic molecules that stimulate trans-basement membrane migration of leukocytes. Concerning this, the proteolytic degradation of the extracellular matrix by MMPs is required to promote extravasation of leukocytes through the EC barrier. Several types of cells in the skin produce MMPs, such as fibroblasts, keratinocytes, and dermal inflammatory cells, which strongly express MMP-9 [66].

For their activities, MMPs play an important role in tissue remodeling, wound healing, and inflammation and could be related to collagen remodeling as a possible cause of the skin manifestations in LS pathology [67]. For example, in tissues affected by vulvar lichen sclerosus, an initially hyperplastic epidermis is followed by keratinocyte death, probably due to an increase in apoptosis processes and the over-expression of MMPs, which promote the catabolism of ECM components and changes in inter-cellular and in cell–ECM connections [67].

In our study, the inhibition of NF-κB induced by MF was associated with the reduction in MIF and MMP9 expression, while OZOILE treatment was not effective on these pathways. Despite this, in our previous studies, OZOILE treatment was proven to be effective in the process of tissue repair of foreskin affected by LS, as demonstrated by the up-regulation of genes encoding for protein involved in tissue regeneration, such as vascular endothelial growth factor (VEGF) and E-cadherin [22,23]. This could be related to the well-recognized connection between NRF2 and VEGF, which was also recently demonstrated by results showing the down-regulation of VEGF in NRF2-silenced endothelial cells [68]. Angiogenesis stimulation by NRF2 could in turn induce the expression of E-cadherin, which is crucial in maintaining epithelial integrity.

In contrast to MF, OZOILE determined a significant increase in the expression levels of SOD2, one of the key targets of NRF2. Beyond the well-known role of SOD2 in the maintenance of cellular oxidative–antioxidant balance and prevention of oxidative stress-related diseases, its preventive role in the inflammation progression of various diseases has also been proven. SOD2 silencing through the CRISPR/Cas9 system caused a higher production of NLRP3 inflammasome components, which might lead to hyper-activation of inflammatory responses.

Additionally, in inflammatory environments like periodontitis, it was shown that the expression of NLRP3 inflammasome components is enhanced in response to deficient SOD2 activity, while caspase-1-IL-1β axis is also magnified, suggesting that SOD2 plays a protective role under inflammatory stimuli [69].

Based on these observations, our data suggest that the activation of the NRF2/SOD2 pathway induced by OZOILE treatment promotes an adaptation to oxidative stress protecting against tissue damage and could contribute to the regulation of inflammatory processes in foreskins affected by LS.

### Limitations of the Study

The limitations of this study include the short treatment duration of only seven days, the small number of recruited patients, and the absence of a placebo group. Therefore, further analyses at later time points with a larger patient population could better illustrate the effects of OZOILE treatment on signaling pathways in foreskin tissues affected by LS. Additionally, the in vivo study cannot establish a direct relationship between OZOILE treatment and the various pathways. To address this, studies involving inhibition or overexpression of the key components of these pathways in cell lines or organoids would be beneficial.

## 5. Conclusions

The results demonstrate that the effects of OZOILE cream treatment, unlike those of corticosteroids, are attributed to the induction of NRF2 signaling rather than the activation of NF-κB, reducing the oxidative stress status. NRF2 has been implicated in different human pathologies and plays a key role in modulating inflammation through multiple mechanisms, including the regulation of redox homeostasis; thus, we hypothesize that the NRF2 pathway could play a key role in the therapeutic mechanism of OZOILE. The different mechanism of action of OZOILE compared to corticosteroids suggests the possible use of OZOILE cream in association with other products for the treatment of inflammatory skin diseases, allowing the use of lower dosages and reducing any side effects of the drugs. However, on the basis of our biochemical data, further studies evaluating the role of NRF2 signaling cascade are required as well as randomized clinical trials addressing the effectiveness of OZOILE for the treatment of LS.

## Figures and Tables

**Table 1:** Primers used for real-time PCR analysis of gene expression.

Target	Primer Sequence 5′ > 3′	
	Forward	Reverse
***NRF2***	CATCACCAGAACACTCAG	CTTCCACTTCAGAATCACT
***p50***	ACACTGGAAGCACGAATGACAGA	CCTCCACCTTCTGCTTGCAA
***p65***	CAGGCGAGAGGAGCACAGATAC	TCCTTTCCTACAAGCTCGTGGG
***MIF***	CGGACAGGGTCTACATCAACTATT	CGGCTCTTAGGCGAAGGT
***MMP2***	TGATCTTGACCAGAATACCATCGA	GGCTTGCGAGGGAAGAAGTT
***MMP9***	GAACCAATCTGTTACGGTCAA	GACTCTCCACGCATCTCT
***SOD2***	TGCTGCTTGTCCAAATCAGG	CACACATCAATCCCCAGCAGT
***β-actin***	TTGTTACAGGAAGTCCCTTGCC	ATGCTATCACCTCCCCTGTGTG

## Data Availability

The raw data supporting the conclusions of this article will be made available by the authors on request.
